# *N*-glycosylation patterns of plasma proteins and immunoglobulin G in chronic obstructive pulmonary disease

**DOI:** 10.1186/s12967-018-1695-0

**Published:** 2018-11-21

**Authors:** Tamara Pavić, Dario Dilber, Domagoj Kifer, Najda Selak, Toma Keser, Đivo Ljubičić, Andrea Vukić Dugac, Gordan Lauc, Lada Rumora, Olga Gornik

**Affiliations:** 1 Faculty of Pharmacy and Biochemistry, University of Zagreb, A. Kovačića 1, 10 000 Zagreb, Croatia; 2 Deparment of Cardiology, County Hospital Čakovec, Čakovec, Croatia; 3 Department of Pulmonology, Clinical Hospital Dubrava, Zagreb, Croatia; 4 Clinical Department for Lung Diseases Jordanovac, University Hospital Centre, Zagreb, Croatia; 5 School of Medicine, University of Zagreb, Zagreb, Croatia; 6 Genos Glycoscience Research Laboratory, Zagreb, Croatia

**Keywords:** *N*-glycosylation, COPD, Biomarkers, Plasma glycoproteins, Immunoglobulin G

## Abstract

**Background:**

Chronic obstructive pulmonary disease (COPD) is a complex condition, whose diagnosis requires spirometric assessment. However, considering its heterogeneity, subjects with similar spirometric parameters do not necessarily have the same functional status. To overcome this limitation novel biomarkers for COPD have been investigated. Therefore, we aimed to explore the potential value of *N*-glycans as COPD biomarkers and to examine the individual variation of plasma protein and immunoglobulin G (IgG) glycosylation profiles in subjects with COPD and healthy controls.

**Methods:**

Both the total plasma protein and IgG *N*-glycome have been profiled in the total of 137 patients with COPD and 95 matching controls from Croatia. Replication cohort consisted of 61 subjects with COPD and 148 controls recruited at another Croatian medical centre.

**Results:**

Plasma protein *N*-glycome in COPD subjects exhibited significant decrease in low branched and conversely, an increase in more complex glycan structures (tetragalactosylated, trisialylated, tetrasialylated and antennary fucosylated glycoforms). We also observed a significant decline in plasma monogalactosylated species, and the same change replicated in IgG glycome. *N*-glycans also showed value in distinguishing subjects in different COPD GOLD stages, where the relative abundance of more complex glycan structures increased as the disease progressed. Glycans also showed statistically significant associations with the frequency of exacerbations and demonstrated to be affected by smoking, which is the major risk factor for COPD development.

**Conclusions:**

This study showed that complexity of glycans associates with COPD, mirroring also the disease severity. Moreover, changes in *N*-glycome associate with exacerbation frequency and are affected by smoking. In general, this study provided new insights into plasma protein and IgG *N*-glycome changes occurring in COPD and pointed out potential novel markers of the disease progression and severity.

**Electronic supplementary material:**

The online version of this article (10.1186/s12967-018-1695-0) contains supplementary material, which is available to authorized users.

## Background

Chronic obstructive pulmonary disease (COPD) is a heterogeneous and complex disease, characterized by lung parenchymal destruction (emphysema), small airways disease (obstructive bronchiolitis), mucociliary dysfunction and chronic airway inflammation [1]. As an important and growing cause of morbidity and mortality, COPD represents a significant burden to the health-care system, with its estimated prevalence of 11.7% (in the adult population) [2]. Moreover, WHO Global Burden of Disease Project predicted COPD to be the third leading cause of death by 2020 [3].

Symptomatology pointing to COPD includes wheeze, dyspnea, chronic cough and sputum production. Establishing a diagnosis of COPD still requires spirometric assessment, regardless of the recent advancements in the COPD management [4]. Spirometry is used to determine the level of airflow limitation, where changes in forced expiratory volume in 1 s (FEV_1_) over time used to serve as a measure of disease progression [5]. Yet, considering COPD heterogeneity, patients with similar FEV_1_ do not necessarily have the same functional status or underlying pathology, making spirometric assessment alone insufficient for thorough characterization of the individual’s status [6]. Because only a weak correlation between FEV_1_, symptoms and impairment of a patient’s health status existed [7], a formal symptomatic assessment was introduced [8]. Also, there was a substantial necessity for measures/biomarkers which would enable reliable patient assessment, management of the treatment and monitoring of the disease progression over shorter periods of time.

To fulfil the aforementioned requirements several potential COPD biomarkers have been investigated recently [9–14]. Roughly, they could be divided into two subgroups—plasma biomarkers originating from lungs, which can also be denoted as local inflammatory markers, and systemic inflammation-related biomarkers. The most investigated ones from the pneumoproteins group include serum club (Clara) cell protein 16 (CC-16) and surfactant protein D (SP-D) [7]. CC-16 showed associations with accelerated decline in lung function [9], while SP-D associated with an increased risk of exacerbations [10], although observed associations were very weak. Since low-grade systemic inflammation marks COPD, it was hypothesized that certain systemic inflammatory biomarkers would associate with some of the disease features, such as exacerbation frequency or mortality risks. There was a strong evidence of an association between fibrinogen and the presence of COPD, frequency of exacerbations and mortality [14]. Thereby, fibrinogen showed association with disease severity but was not able to predict lung function decline. These findings eventually led to the FDA qualification of plasma fibrinogen as a prognostic or an enrichment factor, in addition to standard inclusion/exclusion criteria, in COPD clinical trials with endpoints of COPD exacerbation and/or all-cause mortality [15]. There are also other promising plasma biomarkers for COPD, such as C-reactive protein (CRP) [16] and serum amyloid A protein (SAA) [17], which are acute phase proteins as well. Taking into consideration the importance of inflammatory component of the COPD, several studies were conducted focusing on several inflammatory biomarkers simultaneously. One study monitored individual’s inflammome, i.e. six inflammatory biomarkers [white blood cells count, CRP, interleukin 6 (IL-6), IL-8, fibrinogen and tumor necrosis factor-α (TNF-α)], observing that persistently inflamed subjects had significantly increased mortality and exacerbation frequency compared to non-inflamed ones [18]. Another study showed that simultaneously elevated levels of C-reactive protein (CRP), fibrinogen and leukocyte count were associated with increased risk of exacerbations, even in individuals with milder COPD and in those without previous exacerbations [11].

The inflammatory response, which clearly plays a significant role in COPD pathophysiology, requires fine tuning of many cellular and extracellular processes, such as complement activation, production of cytokines, release of endothelin and the expression of adhesion molecules on leukocytes and the endothelium. The complex arrangement and highly precise regulation of these molecular mechanisms is, among other, dependent on proper *N*-glycosylation of involved molecules [19]. *N*-glycosylation is a co- and posttranslational modification characterized by enzymatic attachment of complex sugar moieties (glycans) to the protein. Correct glycosylation is essential for protein stability and can modulate its function [20]. Previously, it was shown that inflammation associates with disruption of *N*-glycan processing, resulting in aberrant protein glycosylation [21]. Also, *N*-glycans have demonstrated to be very sensitive markers of various disease states [22]. Furthermore, all promising COPD biomarkers, including fibrinogen, are in fact glycoproteins, showing that glycans are involved in nearly all (patho)physiological processes. Glycosylation also modulates function of immunoglobulin G (IgG), a potent and versatile effector member of the immune system. Differential *N*-glycosylation of its fragment crystallizable (Fc) affects IgG effector functions through modified binding affinity to the Fc-receptors (FcγRs), enabling its ability to act as a pro- or anti-inflammatory agent [23]. Therefore, it is also worth exploring IgG glycosylation changes in any disease with the inflammatory component, including COPD. To summarize, glycans could offer a new perspective in the search for COPD biomarkers. Therefore, we aimed to examine the individual variation of plasma protein and IgG glycosylation profiles in subjects with COPD, since this individual variation of *N*-glycosylation in COPD has, to date, never been investigated. Additionally, we aimed to examine glycan changes associated with the disease severity and to explore whether glycan changes show potential as markers of COPD progression. Finally, since smoking represents a major risk factor in COPD aetiology, our goal was and to address the effects of smoking on plasma protein and IgG glycosylation.

## Study population and methods

### Study subjects

All subjects participating in this study were recruited at Croatian medical centres. Discovery cohort consisted of 137 subjects with COPD and 95 matching controls, recruited at University Hospital Centre, Clinical Department for Lung Diseases Jordanovac, Zagreb, Croatia. Replication cohort included 61 subjects with COPD and 148 controls recruited at County Hospital Čakovec, Čakovec, Croatia. Both studies included subjects aged between 40 and 75 years, with the stabile COPD, without exacerbations in the past month, on adequate treatment. COPD was diagnosed according to the 2018 Global Initiative for Chronic Obstructive Lung Disease (GOLD) guidelines, where dyspnea severity was assessed using Modified Medical Research Council (mMRC) scale [4]. Subjects with asthma or other lung diseases, unable to perform lung function tests, with proven coronary artery disease, diabetes mellitus, renal failure, active rheumatoid disease, active autoimmune disease, poorly regulated hypertension, liver disease or malignant disease were excluded. Healthy subjects residing in the same geographic area, with the normal spirometry results were included in the studies as the healthy controls.

Plasma samples were collected in tubes with EDTA as anticoagulant, for the purpose of *N*-glycan profiling. Biochemical tests such as high-sensitivity CRP, fibrinogen, fasting plasma glucose, renal function and lipid profile were performed. Lung function tests were conducted, with output which included FEV_1_, FVC, FEV_1_/FVC. Clinical information on treatment history and smoking exposure (for smokers and ex-smokers) were recorded and anthropometrical measurements collected.

All subjects signed an informed consent and the ethical approvals from the local ethics committees were obtained. The study was conducted in accordance with the Declaration of Helsinki.

### Methods

#### Isolation of IgG from human plasma

IgG was isolated from the plasma samples by affinity chromatography as described previously [24]. In brief, IgG was isolated in a high-throughput manner, using 96-well protein G monolithic plates (BIA Separations, Slovenia), starting from 100 μl of plasma. Plasma was diluted 7× with phosphate buffered saline (PBS; Merck, Germany), applied to the protein G plate and instantly washed. IgG was eluted with 1 ml of 0.1 M formic acid (Merck, Germany) and immediately neutralized with 1 M ammonium bicarbonate (Acros Organics, USA).

#### *N*-glycan release form IgG and total plasma proteins

Isolated IgG samples were dried in a vacuum centrifuge. After drying, IgG was denatured with addition of 30 μl of 1.33% SDS (w/v) (Invitrogen, USA) and by incubation at 65 °C for 10 min. Plasma samples (10 μl) were denatured with the addition of 20 μl of 2% (w/v) SDS (Invitrogen, USA) and by incubation at 65 °C for 10 min. From this point on, the procedure was identical for both IgG and plasma samples. After denaturation, 10 μl of 4% (v/v) Igepal-CA630 (Sigma Aldrich, USA) was added to the samples, and the mixture was shaken 15 min on a plate shaker (GFL, Germany). *N*-glycans were released with the addition of 1.2 U of PNGase F (Promega, USA) and overnight incubation at 37 °C.

#### Fluorescent labelling and HILIC-SPE clean-up of released *N*-glycans

The released *N*-glycans were labelled with 2-aminobenzamide (2-AB). The labelling mixture consisted of 2-AB (19.2 mg/ml; Sigma Aldrich, USA) and 2-picoline borane (44.8 mg/ml; Sigma Aldrich, USA) in dimethyl sulfoxide (Sigma Aldrich, USA) and glacial acetic acid (Merck, Germany) mixture (70:30 v/v). To each sample 25 μl of labelling mixture was added, followed by 2 h incubation at 65 °C. Free label and reducing agent were removed from the samples using hydrophilic interaction liquid chromatography solid-phase extraction (HILIC-SPE). After incubation samples were brought to 96% of acetonitrile (ACN) by adding 700 μl of ACN (J.T. Baker, USA) and applied to each well of a 0.2 μm GHP filter plate (Pall Corporation, USA). Solvent was removed by application of vacuum using a vacuum manifold (Millipore Corporation, USA). All wells were prewashed with 70% ethanol (Sigma-Aldrich, St. Louis, MO, USA) and water, followed by equilibration with 96% ACN. Loaded samples were subsequently washed 5× with 96% ACN. *N*-glycans were eluted with water and stored at − 20 °C until usage.

#### Hydrophilic interaction liquid chromatography of *N*-glycans

Fluorescently labelled *N*-glycans were separated by hydrophilic interaction chromatography on Acquity UPLC H-Class instrument (Waters, USA) consisting of a quaternary solvent manager, sample manager and a fluorescence detector set with excitation and emission wavelengths of 330 and 420 nm, respectively. The instrument was under the control of Empower 2 software, build 2145 (Waters, Milford, USA). Labelled *N*-glycans were separated on a Waters BEH Glycan chromatography column, with 100 mM ammonium formate, pH 4.4, as solvent A and ACN as solvent B. In the case of IgG *N*-glycans, separation method used linear gradient of 75–62% acetonitrile at flow rate of 0.4 ml/min in a 27-min analytical run. For plasma protein *N*-glycans separation method used linear gradient of 70–53% acetonitrile at flow rate of 0.56 ml/min in a 25-min analytical run. The system was calibrated using an external standard of hydrolysed and 2-AB labelled glucose oligomers from which the retention times for the individual glycans were converted to glucose units (GU). Data processing was performed using an automatic processing method with a traditional integration algorithm after which each chromatogram was manually corrected to maintain the same intervals of integration for all the samples. The chromatograms were all separated in the same manner into 24 peaks (IGP1–IGP24) for IgG *N*-glycans and 39 peaks (GP1–GP39) for plasma protein *N*-glycans. Glycan peaks were analysed on the basis of their elution positions and measured in glucose units, then compared to the reference values in the “GlycoStore” database (available at: https://glycostore.org/) for structure assignment [25, 26]. The amount of glycans in each peak was expressed as a percentage of the total integrated area. For IgG glycans, in addition to 24 directly measured glycan traits, nine derived traits were calculated. For plasma glycans, in addition to 39 directly measured glycan traits, 16 derived traits were also calculated. These derived traits average particular glycosylation features, such as galactosylation, fucosylation, bisecting GlcNAc, and sialylation (Table 1).

**Table 1:** Derived glycan traits defined from directly measured glycan peaks

Derived plasma glycan trait	Description	Formula	Example of a glycan structure
LB	Proportion of mono- and biantennary structures in the total plasma *N*-glycome	GP1 + GP2/2 + GP3 + GP4 + GP5 + GP6 + GP8 + GP9 + GP10 + GP11 + GP12 + GP13 + GP14 + GP15 + GP16 + GP17 + GP18 + GP20 + GP21 + GP22 + GP23	
HB	Proportion of tri- and tetraantennary structures in the total plasma *N*-glycome	GP24 + GP25 + GP26 + GP27 + GP28 + GP29 + GP30 + GP31 + GP32 + GP33 + GP34 + GP35 + GP36 + GP37 + GP38 + GP39	
G0	Proportion of agalactosylated structures in the total plasma *N*-glycome	GP1 + GP2/2	
G1	Proportion of monogalactosylated structures in the total plasma *N*-glycome	GP3 + GP4 + GP5 + GP6 + GP12 + GP13	
G2	Proportion of digalactosylated structures in the total plasma *N*-glycome	GP8 + GP9 + GP10 + GP11 + GP14 + GP15 + GP16 + GP17 + GP18 + GP20 + GP21 + GP22 + GP23	
G3	Proportion of trigalactosylated structures in the total plasma *N*-glycome	GP24 + GP25 + GP26 + GP27 + GP28 + GP29 + GP30 + GP31 + GP32 + GP35	
G4	Proportion of tetragalactosylated structures in the total plasma *N*-glycome	GP33 + GP34 + GP36 + GP37 + GP38 + GP39	
S0	Proportion of asialylated structures in the total plasma *N*-glycome	GP1 + GP2/2 + GP3 + GP4 + GP5 + GP6 + GP8 + GP9 + GP10 + GP11	
S1	Proportion of monosialylated structures in the total plasma *N*-glycome	GP12 + GP13 + GP14 + GP15 + GP16 + GP17	
S2	Proportion of disialylated structures in the total plasma *N*-glycome	GP18 + GP20 + GP21 + GP22 + GP23 + GP24 + GP25 + GP26	
S3	Proportion of trisialylated structures in the total plasma *N*-glycome	GP27 + GP28 + GP29 + GP30 + GP31 + GP32 + GP33 + GP34 + GP35	
S4	Proportion of tetrasialylated structures in the total plasma *N*-glycome	GP36 + GP37 + GP38 + GP39	
Bisecting	Proportion of structures containing bisecting GlcNAc in the total plasma *N*-glycome	GP2/2 + GP3 + GP6 + GP9 + GP11 + GP12 + GP15 + GP17 + GP21 + GP23	
OligoMan	Proportion of oligomannosidic structures in the total plasma *N*-glycome	GP2/2 + GP7 + GP19	
CoreF	Proportion of structures containing core fucose in the total plasma *N*-glycome	GP1 + GP2/2 + GP4 + GP5 + GP6 + GP10 + GP11 + GP13 + GP16 + GP17 + GP22 + GP23 + GP29 + GP31	
AntF	Proportion of structures containing antennary fucose in the total plasma *N*-glycome	GP32 + GP35 + GP39	

Derived IgG glycan trait	Description	Formula	Example of a glycan structure
G0	Proportion of agalactosylated structures in the total IgG *N*-glycome	IGP1 + IGP2 + IGP3 + IGP4 + IGP5 + IGP6	
G1	Proportion of monogalactosylated structures in the total IgG *N*-glycome	IGP7 + IGP8 + IGP9 + IGP10 + IGP11 + IGP16	
G2	Proportion of digalactosylated structures in the total IgG *N*-glycome	IGP12 + IGP13 + IGP14 + IGP15 + IGP17 + IGP18 + IGP19 + IGP21 + IGP22 + IGP23 + IGP24	
S0	Proportion of asialylated structures in the total IgG *N*-glycome	IGP1 + IGP2 + IGP3 + IGP4 + IGP5 + IGP6 + IGP7 + IGP8 + IGP9 + IGP10 + IGP11 + IGP12 + IGP13 + IGP14 + IGP15	
S1	Proportion of monosialylated structures in the total IgG *N*-glycome	IGP16 + IGP17 + IGP18 + IGP19	
S2	Proportion of disialylated structures in the total IgG *N*-glycome	IGP21 + IGP22 + IGP23 + IGP24	
Bisecting	Proportion of structures containing bisecting GlcNAc in the total IgG *N*-glycome	IGP3 + IGP6 + IGP10 + IGP11 + IGP13 + IGP15 + IGP19 + IGP22 + IGP24	
OligoMan	Proportion of oligomannosidic structures in the total IgG *N*-glycome	IGP5	
CoreF	Proportion of structures containing core fucose in the total IgG *N*-glycome	IGP1 + IGP4 + IGP6 + IGP8 + IGP9 + IGP10 + IGP11 + IGP14 + IGP15 + IGP16 + IGP18 + IGP19 + IGP23 + IGP24	

GlcNAc: *N*-acetylglucosamine

#### Statistical analysis

All statistical analyses were performed in R programming software (version 3.3.3).

In each separate cohort clinical characteristics were compared between the subjects with COPD and healthy controls using Fisher’s exact test (categorical variables) and Wilcoxon rank-sum test (continuous variables). To remove technical variation, UPLC glycan data were normalised by total chromatogram area, log-transformed and batch corrected using ComBat method (R package “sva”). Derived glycan traits were calculated as a sum of selected directly measured glycan peaks, after data normalisation and batch correction.

Analyses of association between disease status and each glycan trait were modelled using logistic regression, with sex and age defined as covariates. Additionally, analyses of associations between glycan traits (dependent variable) and different clinical features (independent variables) were modelled using a general linear model, adjusted for age and sex. Every analysis was performed for each cohort separately and then combined using an inverse variance-weighted meta-analysis, where cohorts are defined as a random effect (R package “metaphor”). Prior to the analysis all continues variables were transformed to the standard normal distribution by inverse transformation of ranks to normality (R package “GenABEL”), what makes estimated effects comparable between the two cohorts. In the case-control logistic regression, estimated logarithms of odds ratios correspond to the change in  one standard deviation (SD) unit in the glycan abundance. In the linear regression models, estimated coefficients correspond to the change in the glycan levels (expressed in SD units) for positive change of one SD for continuous variables (for example FEV_1_/FVC) or relative to the referent group for categorical variables. False discovery rate was controlled using Benjamini–Hochberg method. p-value < 0.05 was considered significant.

## Results

### Plasma and IgG *N*-glycome in COPD

**Fig. 1:**
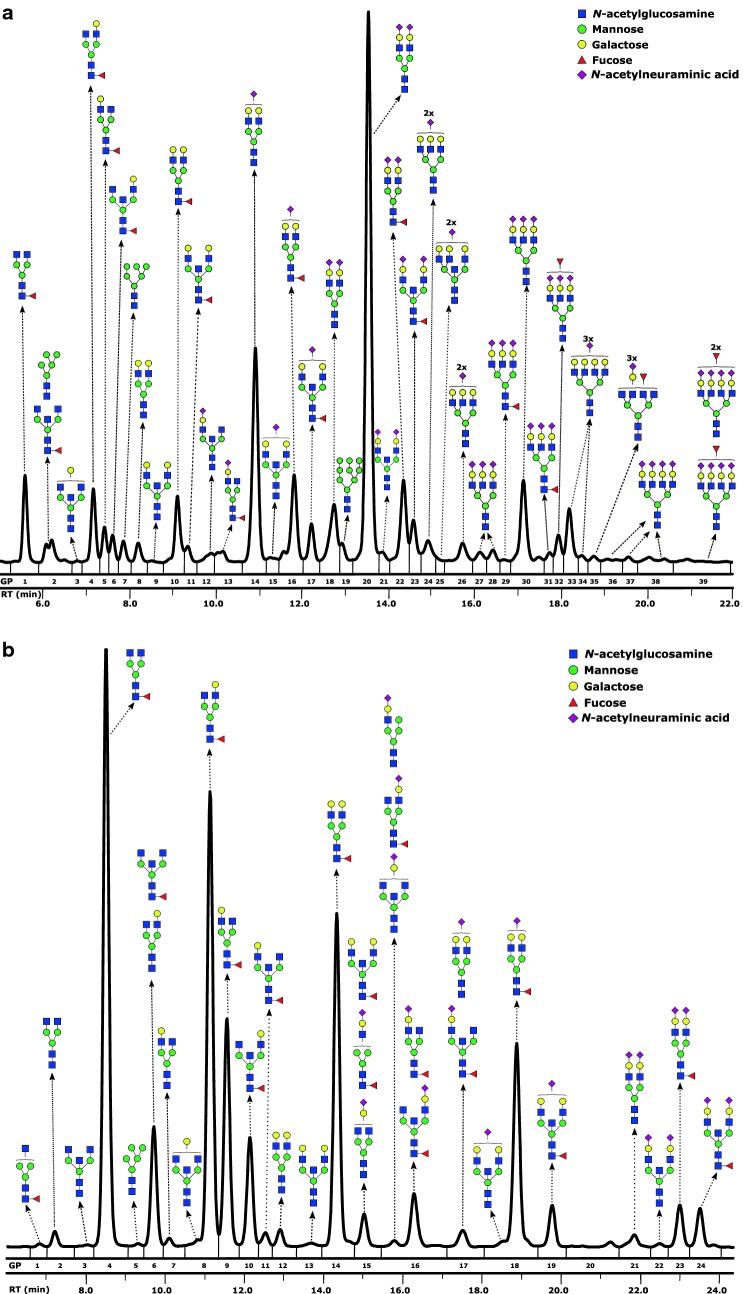
**a** HILIC-UPLC profile of the total *N*-linked glycans released from plasma glycoproteins. Only the most abundant glycoform is presented for each glycan peak. **b** HILIC-UPLC profile of the total *N*-linked glycans released from immunoglobulin G. GP: glycan peak; RT: retention time

Both total plasma protein and IgG *N*-glycome have been profiled in the total of 137 patients with COPD and 95 matching controls from Croatia. Replication cohort consisted of 61 subjects with COPD and 148 controls recruited at another medical centre. Basic description of the cohorts investigated is provided in Table 2. The analysis was conducted by release of *N*-glycans from glycoproteins, their fluorescent labelling and subsequent performance of HILIC-UPLC chromatography. Described approach separates plasma *N*-glycans into 39 (GP1–GP39) and IgG *N*-glycans into 24 (IGP1–IGP24) chromatographic peaks, where each peak was quantified as a percentage of the total *N*-glycome. General description of the glycan structures corresponding to each chromatographic peak is provided in Additional file 1: Table S1. Example of a typical total plasma protein and IgG *N*-glycan chromatographic profile is depicted in Fig. 1. Based on the similar structural features of the analysed glycans, 16 additional derived glycan traits were calculated for plasma protein glycome and nine derived glycan traits were calculated for IgG glycome (Table 1).

**Table 2:** Clinical characteristics of the study participants

	Cohort #1 (discovery)			Cohort #2 (replication)		
	Control	COPD	p	Control	COPD	p
N	95	137		148	61	
Sex						
Males (N)	49 (52%)	86 (63%	0.105	52 (35%)	41 (67%)	< 0.001
Females (N)	46 (48%)	51 (37%)		96 (65%)	20 (33%)	
Age (years)	64 (58–67)	65 (59–72)	0.050	49 (45–57)	63 (59–72)	< 0.001
GOLD classification (N)^a^						
1		0 (0%)			11 (18%)	
2		48 (35%)			23 (38%)	
3		49 (36%)			18 (30%)	
4		40 (29%)			9 (15%)	
A		66 (48%)			13 (21%)	
B		31 (23%)			21 (34%)	
C		7 (5%)			5 (8%)	
D		33 (24%)			22 (36%)	
Exacerbations in the past 12 months (N)						
None		38 (28%)			31 (51%)	
One		59 (43%)			21 (34%)	
Two or more		40 (29%)			9 (15%)	
Smoking (N)						
Non-smokers	48 (51%)	10 (7%)		65 (44%)	5 (8%)	< 0.001
Smokers	47 (49%)	37 (27%)		56 (38%)	43 (70%)	
Ex-smokers	0 (0%)	90 (66%)	< 0.001	27 (18%)	13 (22%)	
hsCRP (mg/l)	2.02 (0.74–2.76)	2.49 (1.27–5.47)	< 0.001	1.10 (0.60–2.50)	2.20 (1.10–5.50)	< 0.001
Fibrinogen (g/l)	3.50 (3.10–3.80)	3.80 (3.40–4.30)	< 0.001	3.20 (2.55–3.70)	3.80 (3.50–4.20)	< 0.001
FVC (l)	3.35 (2.78–4.16)	2.28 (1.81–2.77)	< 0.001	3.77 (3.31–4.66)	3.33 (2.58–3.78)	< 0.001
FEV_1_ (l)	2.60 (2.12–3.17)	1.08 (0.78–1.57)	< 0.001	2.96 (2.60–3.62)	1.49 (1.17–1.92)	< 0.001
FEV_1_/FVC (%)	80.6 (76.9–87.4)	48.2 (41.1–58.2)	< 0.001	79.1 (76.7–82.4)	50.6 (39.3–61.7)	< 0.001

Data are presented as median (interquartile range) for continuous variables and N (%) for categorical variables. Wilcoxon rank-sum test was applied to compare groups for continuous data, while the differences of frequencies for categorical variables were tested using the Fisher’s exact test. The p-value < 0.05 was considered significant

FVC: forced vital capacity; FEV_1_: forced expiratory volume in 1 s; hsCRP: high-sensitivity C-reactive protein

^a^ ABCD assessment tool is based on the Modified Medical Research Council (mMRC) questionnaire

**Fig. 2:**
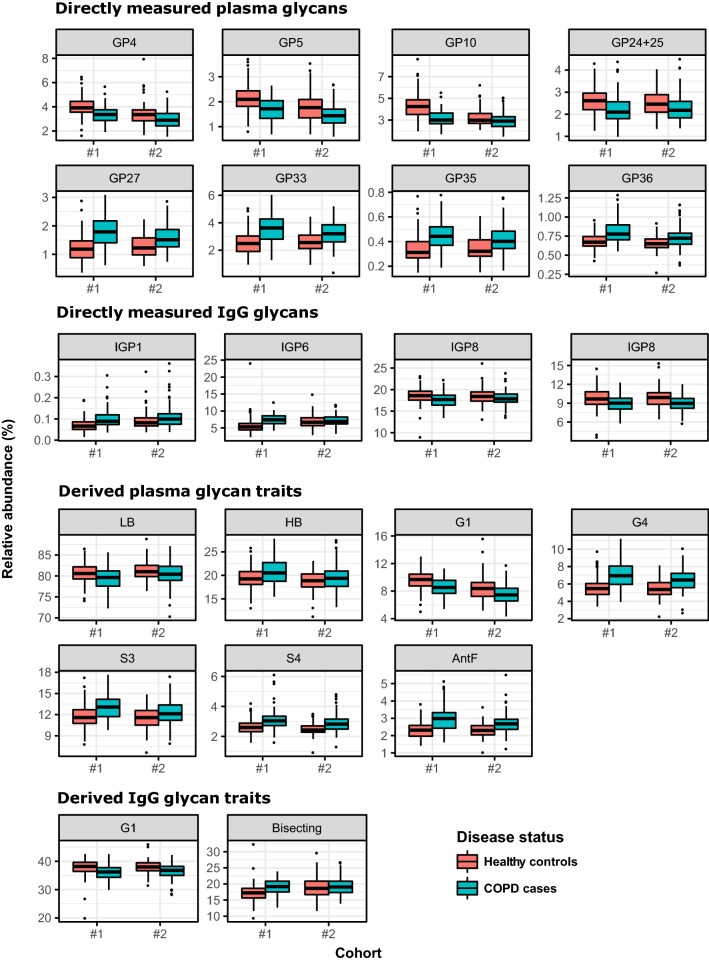
Differences in abundance of plasma protein and IgG glycans between COPD subjects and healthy controls. Differences in glycan abundances are shown as box plots. Each box represents the 25th to 75th percentile. The upper whisker extends from 75th percentile to the values within 1.5 × IQR (where IQR is the inter-quartile range, or distance between the first and third quartiles). The lower whisker extends from 25th percentile to the values within 1.5 × IQR. Data beyond the end of the whiskers are called “outlying” points and are plotted individually. AntF: antennary fucosylation; G1: monogalactosylation; G4: tetragalactosylation; GP: glycan peak; HB: high branching; LB: low branching; S3: trisialylation; S4: tetrasialylation; #1: discovery cohort; #2: replication cohort

Firstly, we tested if glycans can distinguish individuals with COPD from the healthy controls. Hence, we compared directly measured glycan traits between the COPD cases and controls in both, discovery and replication cohort. We found that 16 out of 39 directly measured plasma glycan traits (GPs) and 4 out of 24 IgG traits (IGPs) differ significantly between the groups (Additional file 2: Table S2). Glycans which showed the most pronounced differences between studied groups are presented in Fig. 2.

Next, we examined the performance of derived glycan traits, which average particular glycosylation features (see Table 1 for detailed description of derived traits). We observed that 7 out of 16 derived plasma glycan traits differed significantly between the COPD patients and the controls. In particular, low branched (mono- and biantennary species) and monogalactosylated glycans were significantly lowered in subjects with COPD, and, conversely, high branched (tri- and tetraantennary), more complex glycan species were significantly elevated in COPD (Fig. 2). Of nine derived IgG glycan traits, only monogalactosylation showed to be significantly lowered (adjusted p = 9.45 × 10^−4^) and incidence of bisecting GlcNAc significantly increased in subjects with COPD (adjusted p = 4.89 × 10^−2^) (Additional file 2: Table S2, Fig. 2). Decline in monogalactosylated species in the IgG glycome is consistent with the observations obtained with plasma glycans.

### Glycans as a tool in assessing COPD severity

Sequentially, we wanted to test if glycans are able to distinguish COPD patients in different stages of the disease from the healthy controls. For this purpose, a general linear model was built and case-control meta-analysis was performed on both herein studied cohorts. We evaluated glycan performance using COPD classification described in GOLD 2018 report [4]. GOLD classification includes lung function parameters, but also severity of symptoms and number of exacerbations, where designated number provides information regarding severity of the airflow limitation, while letter referees to symptom burden and risk of exacerbations. The classification of airflow limitation severity is based on spirometric assessment and it divides patients into GOLD stages 1 (mild), 2 (moderate), 3 (severe) and 4 (very severe). Based on assessment of symptoms (herein assessed by mMRC questionnaire) and history of moderate and severe exacerbations, patients are separated into ABCD groups.

**Fig. 3:**
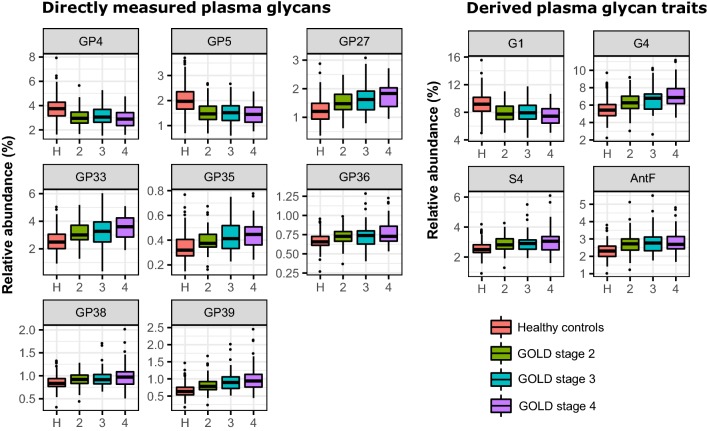
Differences in abundance of plasma protein glycan traits between COPD subjects in different GOLD stages and healthy controls. Only the most significant differences are depicted, resulting from case-control meta-analysis, performed on both herein studied cohorts. Differences in *N*-glycan abundances are shown as box plots. Each box represents the 25th to 75th percentile. Lines inside the boxes represent the median. The upper whisker extends from 75th percentile to the values within 1.5 × IQR (where IQR is the inter-quartile range, or distance between the first and third quartiles). The lower whisker extends from 25th percentile to the values within 1.5 × IQR. Data beyond the end of the whiskers are called “outlying” points and are plotted individually. AntF: antennary fucosylation; G4: tetragalactosylation; GP: glycan peak

Firstly, we investigated associations of plasma glycans with the different stages (GOLD 2–4) of COPD. GOLD stage 1 was omitted from the analysis, since the discovery cohort did not include patients classified as GOLD 1. We observed 42 significant associations of directly measured plasma glycans (GPs) and 23 significant associations of derived plasma glycan traits (Additional file 3: Table S3) with the COPD severity. The strongest associations are depicted in Fig. 3, which clearly demonstrates that changes in the levels of directly measured plasma glycans (GPs) and derived plasma glycan traits mirror the progression of COPD. The most prominent changes were observed in the levels of monogalactosylation, tetragalactosylation, tetrasialylation and antennary fucosylation (Fig. 3), indicating that complexity of glycans increases as COPD advances. Directly measured IgG glycans (IGPs) and derived IgG glycan traits showed poorer performance than plasma glycans in distinguishing patients in the different COPD stages. We observed only 5 significant associations for directly measured IgG glycans and 3 for derived IgG glycan traits. Again, of all IgG glycan traits, monogalactosylation was the feature with the most significant alteration (Additional file 3: Table S3).

Since ABCD assessment tool contributes to the classification of the COPD, we also examined glycan performance in distinguishing patients classified into groups A–D from the healthy controls. Again, we observed significant associations of directly measured glycans and derived glycan traits with the symptom severity expressed through ABCD assessment tool (Additional file 4: Figure S1, Additional file 5: Table S4). In summary, the glycans which showed the most pronounced associations with the symptom severity were basically the same ones which significantly associated with the airflow limitation severity, implying that glycans reflect the general progression of COPD.

**Fig. 4:**
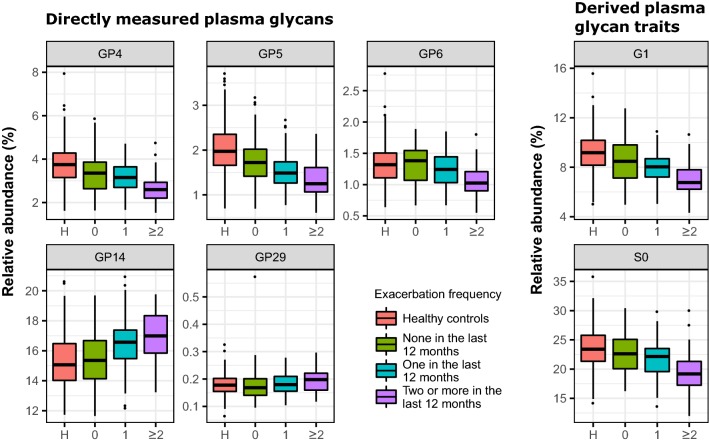
Differences in abundance of plasma protein glycan traits between COPD subjects experiencing various number of exacerbations in the past 12 months and healthy controls. Differences in *N*-glycan abundances are shown as box plots, resulting from case-control meta-analysis, performed on both herein studied cohorts. Each box represents the 25th to 75th percentile. Lines inside the boxes represent the median. The upper whisker extends from 75th percentile to the values within 1.5 × IQR (where IQR is the inter-quartile range, or distance between the first and third quartiles). The lower whisker extends from 25th percentile to the values within 1.5 × IQR. Data beyond the end of the whiskers are called “outlying” points and are plotted individually. G1: monogalactosylation; GP: glycan peak; S0: asialylation

Moderate and severe exacerbation frequency is a part of the ABCD classification as well. Moreover, preventing or diminishing the occurrence of exacerbations is the major therapeutic goal in COPD [4]. For that reason, we examined whether glycans have a value in predicting the frequency of exacerbations and if they could, in this way, potentially assist in their prevention. However, IgG glycans could not predict the exacerbation frequency, while only a few plasma glycans showed statistically significant associations with the exacerbation frequency when patient experienced at least two exacerbations in the past 12 months (Fig. 4, Additional file 6: Table S5). These results indicate only limited potential of the glycans in this regard. However, we would like to point out the observation that the most of the plasma glycans significantly associated with the exacerbation frequency are not the most significant ones in distinguishing COPD itself (with the exception of GP4 and GP5).

### Glycan changes induced by smoking, the major risk factor in aetiology of COPD

**Fig. 5:**
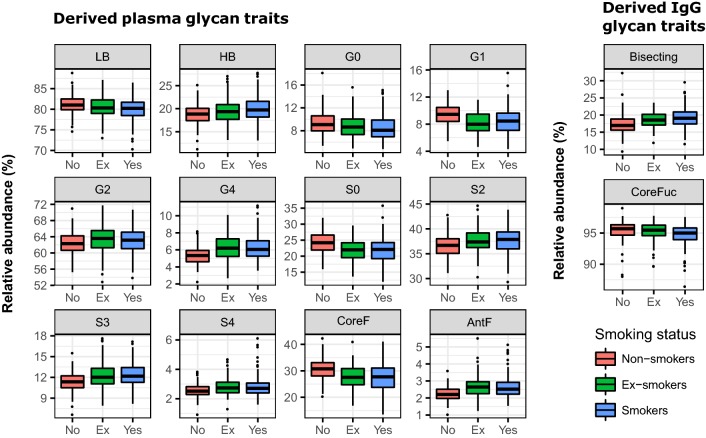
Glycans are affected by smoking. Here are presented differences in abundances of plasma and IgG glycan traits between smokers (pack-year ≥ 10), ex-smokers (quit smoking at least 6 months before study enrolment) and non-smokers. Differences in *N*-glycan abundances are shown as box plots, resulting from case-control meta-analysis, performed on both herein studied cohorts. Each box represents the 25th to 75th percentile. Lines inside the boxes represent the median. The upper whisker extends from 75th percentile to the values within 1.5 × IQR (where IQR is the inter-quartile range, or distance between the first and third quartiles). The lower whisker extends from 25th percentile to the values within 1.5 × IQR. Data beyond the end of the whiskers are called “outlying” points and are plotted individually. AntF: antennary fucosylation; CoreF: core fucosylation; G0: agalactosylation; G1: monogalactosylation; G2: digalactosylation; G4: tetragalactosylation; HB: high branching; LB: low branching; S0: asialylation; S2: disialylation; S3: trisialylation; S4: tetrasialylation

Worldwide, the most commonly encountered risk factor for development of COPD is tobacco smoking [4]. For that reason, we examined effects of smoking on relative abundance of different glycans originating from plasma glycoproteins or IgG alone. We compared changes in glycan levels among three groups of participants, established according to their smoking status. First group consisted of 128 non-smokers, second of 183 smokers, defined as subjects with pack-years ≥ 10 (number of cigarettes per day multiplied by years of smoking/20 cigarettes) and third of 130 ex-smokers, defined as subjects who quit smoking at least 6 months before enrolment in the study. Since many plasma and IgG glycan features (both directly measured and derived) show strong associations with smoking, we restricted our report to derived glycan traits only, to simplify the data presentation. Our analysis revealed that participants who are active smokers have significant decrease of low branched, core fucosylated, agalactosylated, monogalactosylated and asialylated glycoforms originating form plasma (adjusted p-value range: 6.8 × 10^−9^–1.2 × 10^−4^) (Fig. 5, Additional file 7: Table S6). On the other hand, we observed a significant increase of high branching, di- and tetragalactosylated, di-, tri- and tetrasialylated and antennary fucosylated glycoforms in those subjects (adjusted p-value range: 1.7 × 10^−7^–9.5 × 10^−3^). In general, smokers showed a significantly higher abundance of more complex, highly branched glycoforms, and, conversely, a lower abundance of simpler glycan structures in comparison to non-smokers. The same changes were evident when the ex-smokers were compared to the non-smokers, but of a lesser magnitude. For the derived IgG glycan traits, we observed significant increase of glycoforms with bisecting GlcNAc (adjusted p = 1.8 × 10^−7^) and decrease in core fucosylated glycans (adjusted p = 5.5 × 10^−3^), however, only for the smokers and not for the ex-smokers (Fig. 5, Additional file 7: Table S6). Our results revealed that smoking influenced relative abundance of many glycans, significantly affecting the vast majority of the studied derived plasma glycan traits.

## Discussion

To the best of our knowledge, this study is the first one to address individual variation of plasma protein and IgG *N*-glycosylation in chronic obstructive pulmonary disease. Plasma protein glycosylation exhibited several extensive changes, which were more pronounced than the changes occurring in the IgG glycome. We observed that subjects with COPD show a decrease in low branched glycoforms (mono- and biantennary glycans) and conversely, an increase in more complex glycan structures. Also, we revealed a significant reduction in plasma monogalactosylated speciesand this change replicated for IgG glycome as well. Increasing complexity of the plasma glycome in COPD is driven mainly through significant augmentation of relative abundance of tetragalactosylated, tri- and tetrasialylated, and antennary fucosylated glycans. Similar changes in plasma glycome have been marked in several other conditions with inflammatory component, such as type 2 diabetes [27] or low back pain [28], as well as in acute systemic inflammation [21]. The common denominator of all these conditions is the increased branching of the glycans, driven mostly by increase in galactosylated and sialylated species, which the aforementioned studies reported. However, these studies did not monitor the levels of antennary fucosylation, which is one of the glycosylation features with the most pronounced change in COPD patients (next to mono- and tetragalactosylation). Glycan structures bearing antennary fucose can participate in forming of sialyl Lewis-X structures, which have a very important role in the initiation of inflammation [29, 30]. Hence, even according to the pattern of glycosylation changes of the plasma proteins, the inflammatory component has an important role in pathophysiology of COPD. As already stated, COPD is characterized by local inflammatory response in the lungs. Although the disease primarily affects the lungs, it can also produce a substantial systemic response, which is here clearly mirrored through the plasma glycome changes. However, a recent study which examined the associations of COPD with the inflammome (systemic inflammatory network pattern) has demonstrated that only 15% of COPD patients exhibit signs of systemic inflammation, and, moreover, 30% of them did not have any abnormal biomarker of systemic inflammation [18]. This could indicate that the plasma *N*-glycans have a greater sensitivity to the restricted, localized, chronic inflammation than the most of the commonly used serum/plasma biomarkers of inflammation. Furthermore, this could be potentially exploited to monitor the disease progression or patient’s status via plasma glycome alterations. One major drawback we here need to acknowledge, is the fact that we cannot differentiate if the observed plasma glycome changes are in fact the consequence of variation in glycosylation of individual proteins or just the result of their concentration variation. However, we presume that the total plasma protein *N*-glycome reflects an overall trend of glycosylation changes, exhibiting both changes in glycosylation of individual proteins as well as changes in their plasma concentration, resulting with enhanced sensitivity to the pathophysiological events.

The most prominent change of IgG *N*-glycome associating with COPD is a decrease in monogalactosylation. In general, this alteration in IgG glycosylation is thought to decrease its immunosuppressive ability, by enabling recognition and binding of agalactosylated IgG by mannose-binding lectin, and sequential activation of the lectin complement pathway [31]. Decrease in galactosylated glycoforms of IgG was previously reported in a different autoimmune and inflammatory diseases [32, 33], therefore, cannot be considered as a disease-specific marker. Recently, there have been increasing indications of an antigen-specific and, possibly, autoimmune response in COPD. Namely, IgG autoantibodies with avidity for pulmonary epithelium (i.e. HEp-2 epithelial cells, bronchial epithelial cells and endothelial cells) and the potential to mediate cytotoxicity, seem to be prevalent in patients with COPD [34]. Therefore, monitoring IgG glycosylation changes shows prospect in observing the early signs of pathophysiological processes in at-risk subjects.

According to the GOLD initiative definition, COPD is a preventable and treatable disease characterized by persistent respiratory symptoms and airflow limitation [4]. Essentially, there are tools, including particular biomarkers, which can aid in monitoring the patient’s status and disease progression, still, they all show certain limitations. For instance, one of the most extensively used spirometric parameters, FEV_1_, is poorly related to other clinically relevant symptoms (7) and requires a substantial length of observation time for disease progression monitoring (6). For that reason, we examined glycan changes in different disease stages. From our results it is notable that plasma glycan changes reflect the progression of the disease, therefore, could be potentially used to stratify patients according to the disease severity or to monitor the disease progression. The newer GOLD ABCD assessment tool had also put an accent onto patient’s clinical symptoms and exacerbation frequency. In the meanwhile, the prevention of the occurrence of exacerbations became one of the major therapeutic goals in COPD [4], since they are a substantial burden for both patient and the health care system. Exacerbations essentially represent an increase in the inflammation that is present in the stable state, being induced by both infectious and non-infectious causes [35–37]. In some patients, no cause is identified, and presently there are no reliable biomarkers which would predict them [35]. Herein we examined whether glycans have a value in predicting the frequency of exacerbations and observed that monogalactosylated and asialylated plasma glycoforms significantly decreased with the increase of exacerbation frequency. Although glycan changes show potential, their performance was not so powerful to enable a confident prediction of a single annual exacerbation event. However, a recent study [38] has demonstrated that lower levels of IgG1 or IgG2 subclasses resulted in increased risk of exacerbations, where approximately 20% of COPD patients had one or more IgG subclass deficiencies. It would be interesting to analyse subclass-specific glycome in COPD patients to check for associations between glycome composition and exacerbation frequency. Hence, further research would be needed to explore the possibilities of glycan utilisation in this regard.

The last aim of our study was to examine effects of smoking on relative abundances of different glycoforms. The fact that smoking induces changes in glycosylation pattern of various glycoproteins is well known from the previous studies [39, 40]. Herein, smoking resulted in significant increase of IgG glycoforms with bisecting GlcNAc and decrease in core fucosylated glycans, which is consistent with the results from the previous studies [39, 41]. Both increased level of bisecting GlcNAc and decreased level of core fucosylation of IgG glycans coincides with the increased proinflammatory potential of the IgG [42, 43], proving that smoking leads to inflammation [44, 45]. Similarly, the changes in plasma glycome, occurring as a consequence of smoking, also showed inflammation-like pattern—the increase in high branched structures, highly sialylated and antennary fucosylated glycans. These findings somewhat coincide with the previous studies [39, 40], yet, some of them are novel, so they would require further investigation and replication. However, they could be revealing some novel associations, which could help to elucidate the exact mechanisms of studied pathophysiological changes.

## Conclusions

Up to date there was no extensive research conducted which would be focused on investigating the alterations of plasma protein or IgG *N*-glycans in COPD. Ever-growing evidence of the *N*-glycosylation importance is demonstrating that the glycan parts of glycoprotein are as essential as the protein itself, since they are modifying its structure, properties and function [20]. This clearly suggests that the glycans should not be neglected when investigating underlying molecular mechanisms of any studied condition. Also, the possibilities should be explored whether glycans could be useful as predictive or stratifying tools, since they do show the great sensitivity of change in response to the various genetic and environmental influences. This study demonstrated that glycans are able to differentiate COPD patients from the healthy individuals and can distinguish patients in the different stages of the disease, which could potentially be exploited to monitor the disease progression, patient’s status, and even response to treatment. Moreover, glycan profiles showed to be affected by smoking, which is the major risk factor for COPD development, demonstrating that they could help in detecting pathophysiological changes at their earliest. However, due to the cross-sectional design of our study we cannot speculate on causality of those glycan changes, nor can we monitor long-term outcomes. In conclusion, this study provided new insights into glycan changes occurring in COPD and indicated new prospects in the search for markers of COPD progression and severity.

## Additional files

**Additional file 1: Table S1.** General description of directly measured plasma protein (GPs) and IgG (IGPs) glycan traits, showing the corresponding glycan structures.

**Additional file 2: Table S2.** Associations of the studied glycan traits with the disease status (COPD cases vs healthy controls). Just the statistically significant associations are presented, resulting from case-control meta-analysis. Glycan data were adjusted for age and sex, and corrected for multiple comparisons (Benjamini–Hochberg method).

**Additional file 3: Table S3.** Associations of glycan traits with the COPD severity (cases in different GOLD stages of COPD vs healthy controls). Just the glycan traits with statistically significant associations are presented, resulting from case-control meta-analysis. Glycan data were adjusted for age and sex, and corrected for multiple comparisons (Benjamini-Hochberg method).

**Additional file 4: Figure S1.** Differences in abundance of plasma protein glycan traits between COPD subjects classified into ABCD groups and healthy controls. Differences are shown as box plots, resulting from case-control meta-analysis, performed on both herein studied cohorts. Each box represents the 25th to 75th percentile. Lines inside the boxes represent the median. The upper whisker extends from 75th percentile to the values within 1.5 x IQR (where IQR is the inter-quartile range, or distance between the first and third quartiles). The lower whisker extends from 25th percentile to the values within 1.5 x IQR. Data beyond the end of the whiskers are called “outlying” points and are plotted individually.

**Additional file 5: Table S4.** Associations of glycan traits with the symptom severity of the COPD (cases in different ABCD groups vs healthy controls). Just the glycan traits with statistically significant associations are presented, resulting from case-control meta-analysis. Glycan data were adjusted for age and sex, and corrected for multiple comparisons (Benjamini-Hochberg method).

**Additional file 6: Table S5.** Associations of plasma glycan traits with the exacerbation frequency (cases with different occurrence of exacerbation events vs healthy controls). Just the glycan traits with statistically significant associations are presented, resulting from case-control meta-analysis. Glycan data were adjusted for age and sex, and corrected for multiple comparisons (Benjamini-Hochberg method).

**Additional file 7: Table S6.** Associations of plasma and IgG glycan traits with the smoking status (smokers / ex-smokers vs non-smokers). Just the glycan traits with statistically significant associations are presented, resulting from case-control meta-analysis. Glycan data were adjusted for age and sex, and corrected for multiple comparisons (Benjamini–Hochberg method).

## Abbreviations

2-AB: 2-aminobenzamide
ACN: acetonitrile
AntF: antennary fucosylation
CC-16: club (Clara) cell protein 16
CRP: C-reactive protein
COPD: chronic obstructive pulmonary disease
CoreF: core fucosylation
Fc: fragment crystallizable
FcγR: Fcγ receptor
FEV_1_: forced expiratory volume in one second
FUT: fucosyltransferase
FVC: forced vital capacity
GlcNAc: *N*-acetylglucosamine
GOLD: Global Initiative for Chronic Obstructive Lung Disease
GP: glycan peak
GU: glucose unit
G0: agalactosylation
G1: monogalactosylation
G2: digalactosylation
G3: trigalactosylation
G4: tetragalactosylation
HB: high branching
HILIC: hydrophilic interaction liquid chromatography
IgG: immunoglobulin G
IL: interleukin
IQR: interquartile range
LB: low branching
mMRC: Modified Medical Research Council questionnaire
OligoMann: oligomannosylation
PBS: phosphate buffered saline
RT: retention time
SAA: serum amyloid A protein
SD: standard deviation
SDS: sodium dodecyl sulfate
SP-D: surfactant protein D
SPE: solid-phase extraction
S0: asialylation
S1: monosialylation
S2: desialylation
S3: trisialylation
S4: tetrasialylation
SE: standard error
TNF-α: tumor necrosis factor-α
UPLC: ultra-performance liquid chromatography
WHO: World Health Organization
