# Supplementary material for: N-glycosylation patterns of plasma proteins and immunoglobulin G in chronic obstructive pulmonary disease
Source: J Transl Med. 2018 Nov 21;16:323. doi: 10.1186/s12967-018-1695-0 (PMC6249776; doi:10.1186/s12967-018-1695-0)
Supplement: Supplementary file 2 — Additional file 2: Table S2. Associations of the studied glycan traits with the disease status (COPD cases vs healthy controls). Just the statistically significant associations are presented, resulting from case-control meta-analysis. Glycan data were adjusted for age and sex, and corrected for multiple comparisons (Benjamini–Hochberg method). [file 12967_2018_1695_MOESM2_ESM.docx]

**Additional files**

**N-glycosylation patterns of plasma proteins and immunoglobulin G in chronic obstructive pulmonary disease**

Tamara Pavić^1^, Dario Dilber^2^, Domagoj Kifer^1^, Najda Selak^1^, Toma Keser^1^, Đivo Ljubičić^3^, Andrea Vukić Dugac^4^, Gordan Lauc^1,5^, Lada Rumora^1^, Olga Gornik^1^

^1^Faculty of Pharmacy and Biochemistry, University of Zagreb, Zagreb, Croatia

^2^County Hospital Čakovec, Deparment of Cardiology, Čakovec, Croatia

^3^Clinical Hospital Dubrava, Department of Pulmonology, Zagreb, Croatia

^4^University Hospital Centre, Clinical Department for Lung Diseases Jordanovac, Zagreb, Croatia

^5^Genos Glycoscience Research Laboratory, Zagreb, Croatia

Additional file 2: Table S2. Associations of the studied glycan traits with the disease status (COPD cases vs healthy controls). Just the statistically significant associations are presented, resulting from case-control meta-analysis. Glycan data were adjusted for age and sex, and corrected for multiple comparisons (Benjamini-Hochberg method).*

| ***Origin*** | ***Glycan*** | ***Description⁑,§*** | ***Beta*** | ***SE*** | ***Meta-analysis p-value*** | ***Meta-analysis adjusted p-value*** |
| --- | --- | --- | --- | --- | --- | --- |
| plasma | GP33 | relative abundance of A4G4S3 | 0.6039 | 0.1002 | 1.65E-09 | 8.77E-08 |
| plasma | GP27 | relative abundance of A3G3S3 | 0.5791 | 0.0992 | 5.40E-09 | 1.43E-07 |
| plasma | GP35 | relative abundance of A4FG3S3 | 0.5568 | 0.1038 | 8.14E-08 | 1.08E-06 |
| plasma | GP36 | relative abundance of A4G4S4 | 0.5268 | 0.1048 | 4.93E-07 | 5.23E-06 |
| plasma | GP4 | relative abundance of of FA2[6]G1 | -0.5182 | 0.1082 | 1.66E-06 | 1.47E-05 |
| plasma | GP10 | relative abundance of FA2G2 | -0.4612 | 0.0999 | 3.91E-06 | 2.96E-05 |
| plasma | GP5 | relative abundance of of FA2[3]G1 | -0.6000 | 0.1372 | 1.23E-05 | 8.14E-05 |
| plasma | GP24_GP25 | relative abundance of of A3G3S2 and A3BG3S2 | -0.4042 | 0.1015 | 6.87E-05 | 3.31E-04 |
| plasma | GP28 | relative abundance of A3G3S3 | -0.3765 | 0.1036 | 2.79E-04 | 1.14E-03 |
| plasma | GP39 | relative abundance of A4FG4S4 and A4F2G4S4 | 0.6742 | 0.1938 | 5.04E-04 | 1.91E-03 |
| plasma | GP13 | relative abundance of FA2G1S1 | -0.3656 | 0.1065 | 5.95E-04 | 2.10E-03 |
| plasma | GP31 | relative abundance of FA3G3S3 | -0.3370 | 0.1033 | 1.11E-03 | 3.68E-03 |
| plasma | GP16 | relative abundance of FA2G2S1 | -0.2997 | 0.1057 | 4.59E-03 | 1.35E-02 |
| plasma | GP30 | relative abundance of A3G3S3 | -0.2948 | 0.1052 | 5.09E-03 | 1.42E-02 |
| plasma | GP19 | relative abundance of M9 | -0.2880 | 0.1053 | 6.24E-03 | 1.65E-02 |
| plasma | GP26 | relative abundance of A3G3S2 | -0.2591 | 0.1077 | 1.61E-02 | 3.89E-02 |
| IgG | IGP9 | relative abundance of of FA2[3]G1 | -0.5213 | 0.1095 | 1.95E-06 | 6.42E-05 |
| IgG | IGP1 | relative abundance of of FA1 | 0.3467 | 0.1103 | 1.67E-03 | 1.84E-02 |
| IgG | IGP6 | relative abundance of of FA2B | 0.3166 | 0.1148 | 5.83E-03 | 4.81E-02 |
| IgG | IGP8 | relative abundance of of FA2[6]G1 | -0.2930 | 0.1100 | 7.70E-03 | 4.89E-02 |
| ***Origin*** | ***Derived trait*** | ***Description*** | ***Beta*** | ***SE*** | ***Meta-analysis p-value*** | ***Meta-analysis adjusted p-value*** |
| plasma | G4 | Tetragalactosylation | 0.6518 | 0.1189 | 4.17E-08 | 7.37E-07 |
| plasma | G1 | Monogalactosylation | -0.5338 | 0.1308 | 4.51E-05 | 2.66E-04 |
| plasma | AntF | Antennary fucosylation | 0.5883 | 0.1461 | 5.67E-05 | 3.00E-04 |
| plasma | S3 | Trisialylation | 0.4004 | 0.1086 | 2.27E-04 | 1.00E-03 |
| plasma | S4 | Tetrasialylation | 0.5677 | 0.1871 | 2.41E-03 | 7.52E-03 |
| plasma | HB | High branching | 0.2688 | 0.1097 | 1.42E-02 | 3.59E-02 |
| plasma | LB | Low branching | -0.2623 | 0.1099 | 1.70E-02 | 3.92E-02 |
| IgG | G1 | Monogalactosylation | -0.4340 | 0.1079 | 5.73E-05 | 9.45E-04 |
| IgG | Bisecting | Bisecting GlcNAc | 0.3176 | 0.1214 | 8.89E-03 | 4.89E-02 |

*beta - standardized regression coefficient; GP – plasma glycan peak; IGP – IgG glycan peak; SE- standard error

⁑ for the description of glycan peaks only the most abundant glycoform within the peak is listed

§ for the explanation of glycan structure abbreviations see Additional file 1: Table A1
