# Supplementary material for: N-glycosylation patterns of plasma proteins and immunoglobulin G in chronic obstructive pulmonary disease
Source: J Transl Med. 2018 Nov 21;16:323. doi: 10.1186/s12967-018-1695-0 (PMC6249776; doi:10.1186/s12967-018-1695-0)
Supplement: Supplementary file 3 — Additional file 3: Table S3. Associations of glycan traits with the COPD severity (cases in different GOLD stages of COPD vs healthy controls). Just the glycan traits with statistically significant associations are presented, resulting from case-control meta-analysis. Glycan data were adjusted for age and sex, and corrected for multiple comparisons (Benjamini-Hochberg method). [file 12967_2018_1695_MOESM3_ESM.docx]

Additional file 3: Table S3 Associations of glycan traits with the COPD severity (cases in different GOLD stages of COPD vs healthy controls). Just the glycan traits with statistically significant associations are presented, resulting from case-control meta-analysis. Glycan data were adjusted for age and sex, and corrected for multiple comparisons (Benjamini-Hochberg method).*

| ***Origin*** | ***GOLD classification*** | ***Glycan*** | ***Beta*** | ***SE*** | ***Meta-analysis p-value*** | ***Meta-analysis adjusted p-value*** |
| --- | --- | --- | --- | --- | --- | --- |
| plasma | 4 | GP39 | 1.0021 | 0.1498 | 2.26E-11 | 3.59E-09 |
| plasma | 3 | GP39 | 0.8146 | 0.1350 | 1.58E-09 | 8.36E-08 |
| plasma | 4 | GP27 | 0.9014 | 0.1525 | 3.38E-09 | 1.34E-07 |
| plasma | 4 | GP33 | 0.9014 | 0.1539 | 4.67E-09 | 1.48E-07 |
| plasma | 3 | GP4 | -0.7332 | 0.1441 | 3.63E-07 | 7.71E-06 |
| plasma | 4 | GP35 | 0.8073 | 0.1591 | 3.88E-07 | 7.71E-06 |
| plasma | 2 | GP5 | -0.6905 | 0.1408 | 9.40E-07 | 1.66E-05 |
| plasma | 4 | GP5 | -0.7717 | 0.1593 | 1.28E-06 | 1.85E-05 |
| plasma | 4 | GP38 | 0.7530 | 0.1595 | 2.33E-06 | 2.86E-05 |
| plasma | 4 | GP4 | -0.7577 | 0.1642 | 3.93E-06 | 4.47E-05 |
| plasma | 4 | GP36 | 0.7258 | 0.1605 | 6.12E-06 | 5.72E-05 |
| plasma | 3 | GP35 | 0.6045 | 0.1428 | 2.32E-05 | 1.90E-04 |
| plasma | 3 | GP33 | 0.5766 | 0.1379 | 2.89E-05 | 2.19E-04 |
| plasma | 3 | GP19 | -0.5763 | 0.1428 | 5.43E-05 | 3.76E-04 |
| plasma | 3 | GP24+25 | -0.5567 | 0.1391 | 6.30E-05 | 4.18E-04 |
| plasma | 3 | GP27 | 0.5394 | 0.1364 | 7.69E-05 | 4.89E-04 |
| plasma | 3 | GP36 | 0.5350 | 0.1445 | 2.15E-04 | 1.26E-03 |
| plasma | 2 | GP10 | -0.4946 | 0.1351 | 2.51E-04 | 1.38E-03 |
| plasma | 2 | GP33 | 0.4924 | 0.1344 | 2.49E-04 | 1.38E-03 |
| plasma | 2 | GP13 | -0.5799 | 0.1592 | 2.69E-04 | 1.43E-03 |
| plasma | 2 | GP4 | -0.5420 | 0.1493 | 2.82E-04 | 1.44E-03 |
| plasma | 4 | GP10 | -0.5679 | 0.1568 | 2.92E-04 | 1.45E-03 |
| plasma | 3 | GP28 | -0.5143 | 0.1423 | 3.01E-04 | 1.45E-03 |
| plasma | 3 | GP13 | -0.5227 | 0.1449 | 3.10E-04 | 1.45E-03 |
| plasma | 3 | GP4 | -0.5243 | 0.1483 | 4.06E-04 | 1.84E-03 |
| plasma | 2 | GP32 | 0.5160 | 0.1463 | 4.19E-04 | 1.85E-03 |
| plasma | 2 | GP27 | 0.4543 | 0.1329 | 6.32E-04 | 2.64E-03 |
| plasma | 2 | GP36 | 0.4616 | 0.1411 | 1.07E-03 | 3.94E-03 |
| plasma | 4 | GP26 | -0.5182 | 0.1608 | 1.27E-03 | 4.38E-03 |
| plasma | 3 | GP30 | -0.4557 | 0.1440 | 1.56E-03 | 5.06E-03 |
| plasma | 3 | GP38 | 0.5138 | 0.1622 | 1.53E-03 | 5.06E-03 |
| plasma | 2 | GP20+21 | 0.4597 | 0.1467 | 1.73E-03 | 5.49E-03 |
| plasma | 3 | GP10 | -0.4041 | 0.1390 | 3.65E-03 | 1.14E-02 |
| plasma | 2 | GP38 | 0.4098 | 0.1415 | 3.77E-03 | 1.15E-02 |
| plasma | 3 | GP7 | -0.4218 | 0.1501 | 4.96E-03 | 1.49E-02 |
| plasma | 2 | GP35 | 0.3901 | 0.1393 | 5.10E-03 | 1.50E-02 |
| plasma | 4 | GP28 | -0.4260 | 0.1574 | 6.79E-03 | 1.96E-02 |
| plasma | 3 | GP26 | -0.3851 | 0.1459 | 8.29E-03 | 2.27E-02 |
| plasma | 4 | GP31 | -0.5733 | 0.2242 | 1.06E-02 | 2.84E-02 |
| plasma | 4 | GP19 | -0.3884 | 0.1572 | 1.35E-02 | 3.51E-02 |
| plasma | 4 | GP6 | -0.4205 | 0.1723 | 1.47E-02 | 3.70E-02 |
| plasma | 2 | GP16 | -0.3706 | 0.1578 | 1.89E-02 | 4.62E-02 |
| IgG | 4 | IGP1 | 0.8647 | 0.1608 | 7.59E-08 | 7.51E-06 |
| IgG | 2 | IGP9 | -0.6492 | 0.1479 | 1.13E-05 | 3.72E-04 |
| IgG | 3 | IGP9 | -0.5720 | 0.1436 | 6.76E-05 | 1.67E-03 |
| IgG | 4 | IGP9 | -0.5556 | 0.1643 | 7.22E-04 | 1.20E-02 |
| IgG | 4 | IGP8 | -0.5000 | 0.1657 | 2.56E-03 | 3.16E-02 |
| ***Origin*** | ***GOLD classification*** | ***Derived glycan trait*** | ***Beta*** | ***SE*** | ***Meta-analysis p-value*** | ***Meta-analysis adjusted p-value*** |
| plasma | 4 | G4 | 0.9951 | 0.1543 | 1.12E-10 | 8.90E-09 |
| plasma | 4 | S4 | 0.8951 | 0.1577 | 1.38E-08 | 3.66E-07 |
| plasma | 3 | AntF | 0.6857 | 0.1409 | 1.14E-06 | 1.81E-05 |
| plasma | 3 | G4 | 0.6583 | 0.1386 | 2.05E-06 | 2.72E-05 |
| plasma | 3 | S4 | 0.6543 | 0.1427 | 4.57E-06 | 4.84E-05 |
| plasma | 4 | G1 | -0.7425 | 0.1633 | 5.48E-06 | 5.44E-05 |
| plasma | 4 | AntF | 0.6900 | 0.1573 | 1.16E-05 | 1.02E-04 |
| plasma | 3 | G1 | -0.6235 | 0.1476 | 2.39E-05 | 1.90E-04 |
| plasma | 2 | G1 | -0.5907 | 0.1441 | 4.16E-05 | 3.01E-04 |
| plasma | 2 | G4 | 0.5203 | 0.1352 | 1.19E-04 | 7.26E-04 |
| plasma | 4 | S3 | 0.5776 | 0.1659 | 5.00E-04 | 2.15E-03 |
| plasma | 2 | S0 | -0.4966 | 0.1465 | 6.99E-04 | 2.74E-03 |
| plasma | 3 | S0 | -0.5077 | 0.1499 | 7.05E-04 | 2.74E-03 |
| plasma | 4 | S0 | -0.5605 | 0.1655 | 7.07E-04 | 2.74E-03 |
| plasma | 2 | AntF | 0.6048 | 0.1803 | 7.96E-04 | 3.01E-03 |
| plasma | 2 | S4 | 0.4658 | 0.1433 | 1.15E-03 | 4.17E-03 |
| plasma | 2 | S3 | 0.4705 | 0.1458 | 1.25E-03 | 4.38E-03 |
| plasma | 2 | CoreF | -0.4707 | 0.1463 | 1.30E-03 | 4.39E-03 |
| plasma | 4 | HB | 0.4456 | 0.1666 | 7.48E-03 | 2.12E-02 |
| plasma | 4 | LB | -0.4436 | 0.1668 | 7.82E-03 | 2.18E-02 |
| plasma | 3 | OligoMann | -0.3786 | 0.1487 | 1.09E-02 | 2.89E-02 |
| plasma | 3 | CoreF | -0.3687 | 0.1498 | 1.38E-02 | 3.55E-02 |
| plasma | 2 | HB | 0.3546 | 0.1471 | 1.59E-02 | 3.95E-02 |
| IgG | 4 | G1 | -0.7349 | 0.1613 | 5.20E-06 | 2.57E-04 |
| IgG | 2 | Bisecting | 0.4812 | 0.1425 | 7.30E-04 | 1.20E-02 |
| IgG | 3 | G1 | -0.4657 | 0.1455 | 1.37E-03 | 1.93E-02 |

*AntF – antennary fucosylation; beta - standardized regression coefficient; COPD – chronic obstructive pulmonary disease; CoreF – core fucosylation; G1 – monogalactosylation; G4 – tetragalactosylation; GOLD – Global Initiative for Chronic Obstructive Lung Disease; GP – plasma glycan peak; HB – high branching; IGP – IgG glycan peak; LB – low branching; OligoMann – oligomannosylation; S0 – asialylation; S3 – trisialylation; S4 – tetrasialylation; SE- standard error.
