# Supplementary material for: N-glycosylation patterns of plasma proteins and immunoglobulin G in chronic obstructive pulmonary disease
Source: J Transl Med. 2018 Nov 21;16:323. doi: 10.1186/s12967-018-1695-0 (PMC6249776; doi:10.1186/s12967-018-1695-0)
Supplement: Supplementary file 4 — Additional file 4: Figure S1. Differences in abundance of plasma protein glycan traits between COPD subjects classified into ABCD groups and healthy controls. Differences are shown as box plots, resulting from case-control meta-analysis, performed on both herein studied cohorts. Each box represents the 25th to 75th percentile. Lines inside the boxes represent the median. The upper whisker extends from 75th percentile to the values within 1.5 x IQR (where IQR is the inter-quartile range, or distance between the first and third quartiles). The lower whisker extends from 25th percentile to the values within 1.5 x IQR. Data beyond the end of the whiskers are called “outlying” points and are plotted individually. [file 12967_2018_1695_MOESM4_ESM.docx]

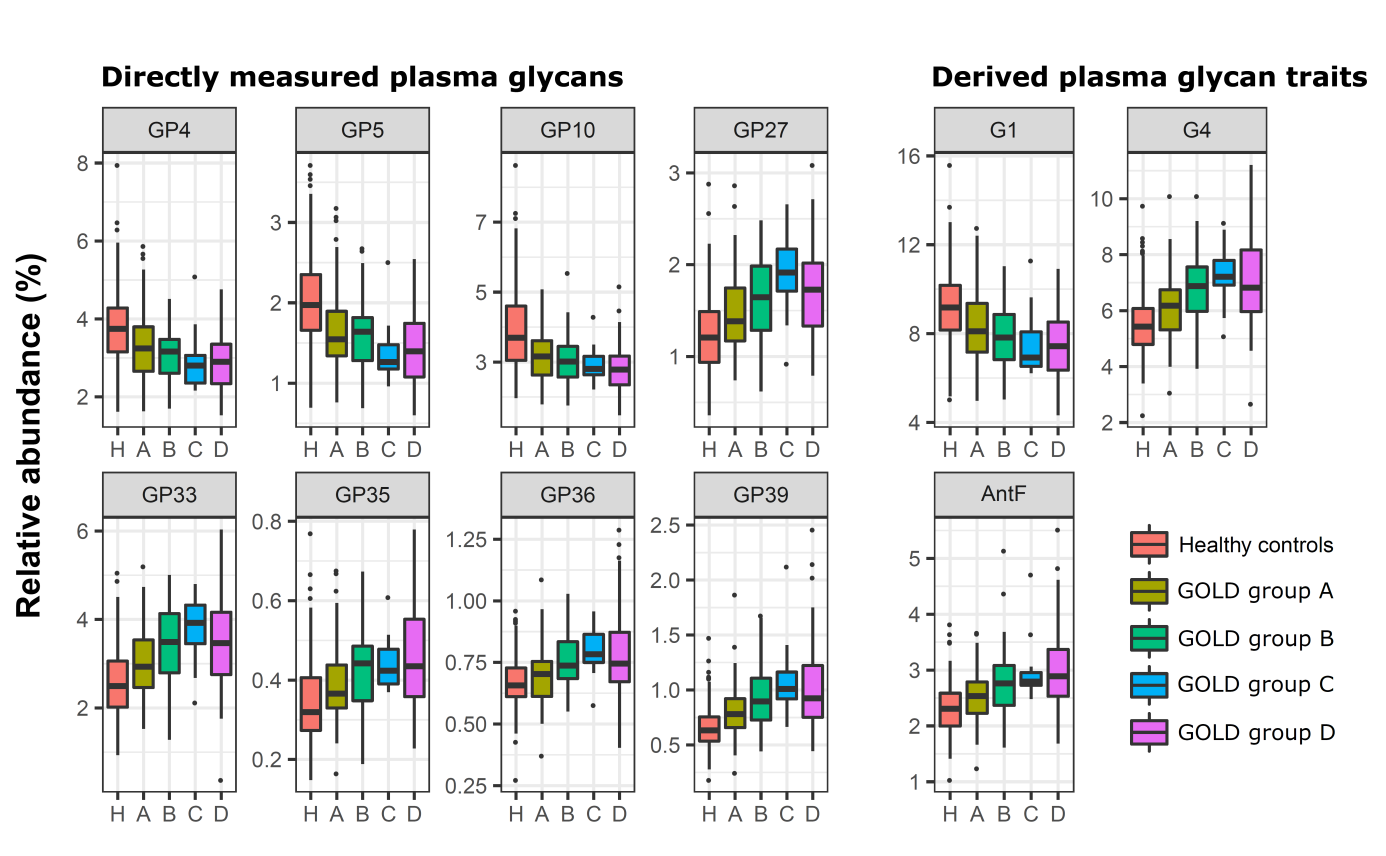


Additional file 4: Figure S1 Differences in abundance of plasma protein glycan traits between COPD subjects classified into ABCD groups and healthy controls. Differences are shown as box plots, resulting from case-control meta-analysis, performed on both herein studied cohorts. Each box represents the 25th to 75th percentile. The upper whisker extends from 75th percentile to the values within 1.5 x IQR (where IQR is the inter-quartile range, or distance between the first and third quartiles). The lower whisker extends from 25th percentile to the values within 1.5 x IQR. Data beyond the end of the whiskers are called "outlying" points and are plotted individually. AntF – antennary fucosylation; G1 – monogalactosylation; G4 – tetragalactosylation; GP – plasma glycan peak.
