# Supplementary material for: N-glycosylation patterns of plasma proteins and immunoglobulin G in chronic obstructive pulmonary disease
Source: J Transl Med. 2018 Nov 21;16:323. doi: 10.1186/s12967-018-1695-0 (PMC6249776; doi:10.1186/s12967-018-1695-0)
Supplement: Supplementary file 5 — Additional file 5: Table S4. Associations of glycan traits with the symptom severity of the COPD (cases in different ABCD groups vs healthy controls). Just the glycan traits with statistically significant associations are presented, resulting from case-control meta-analysis. Glycan data were adjusted for age and sex, and corrected for multiple comparisons (Benjamini-Hochberg method). [file 12967_2018_1695_MOESM5_ESM.docx]

Additional file 5: Table S4. Associations of glycan traits with the symptom severity of the COPD (cases in different ABCD groups vs healthy controls). Just the glycan traits with statistically significant associations are presented, resulting from case-control meta-analysis. Glycan data were adjusted for age and sex, and corrected for multiple comparisons (Benjamini-Hochberg method).*

| ***Origin*** | ***ABCD group based on mMRC*** | ***Glycan*** | ***Beta*** | ***SE*** | ***Meta-analysis p-value*** | ***Meta-analysis adjusted p-value*** |
| --- | --- | --- | --- | --- | --- | --- |
| plasma | D | GP39 | 0.9375 | 0.1445 | 8.74E-11 | 1.85E-08 |
| plasma | D | GP4 | -0.9173 | 0.1536 | 2.34E-09 | 1.11E-07 |
| plasma | D | GP5 | -0.9451 | 0.1611 | 4.43E-09 | 1.57E-07 |
| plasma | D | GP35 | 0.8418 | 0.1500 | 2.01E-08 | 6.09E-07 |
| plasma | D | GP10 | -0.7697 | 0.1433 | 7.88E-08 | 2.09E-06 |
| plasma | D | GP27 | 0.7491 | 0.1430 | 1.61E-07 | 3.79E-06 |
| plasma | D | GP33 | 0.7313 | 0.1443 | 4.05E-07 | 8.59E-06 |
| plasma | D | GP36 | 0.7282 | 0.1509 | 1.39E-06 | 2.68E-05 |
| plasma | D | GP29 | 0.5741 | 0.1609 | 3.61E-04 | 3.64E-03 |
| plasma | D | GP19 | -0.4254 | 0.1535 | 5.57E-03 | 2.51E-02 |
| plasma | D | GP24 + GP25 | -0.4525 | 0.1699 | 7.72E-03 | 3.09E-02 |
| plasma | C | GP27 | 1.0792 | 0.2729 | 7.66E-05 | 1.08E-03 |
| plasma | C | GP5 | -1.0861 | 0.2895 | 1.75E-04 | 2.19E-03 |
| plasma | C | GP39 | 1.1031 | 0.3050 | 2.98E-04 | 3.24E-03 |
| plasma | C | GP33 | 0.9661 | 0.2757 | 4.57E-04 | 4.22E-03 |
| plasma | C | GP36 | 0.9627 | 0.2887 | 8.52E-04 | 6.08E-03 |
| plasma | C | GP28 | -0.9025 | 0.2886 | 1.76E-03 | 1.04E-02 |
| plasma | C | GP31 | -0.8718 | 0.2860 | 2.30E-03 | 1.19E-02 |
| plasma | C | GP8 | 0.8377 | 0.3071 | 6.38E-03 | 2.71E-02 |
| plasma | C | GP13 | -0.7864 | 0.2951 | 7.70E-03 | 3.09E-02 |
| plasma | C | GP4 | -0.8300 | 0.3255 | 1.08E-02 | 3.94E-02 |
| plasma | C | GP30 | -0.7355 | 0.2938 | 1.23E-02 | 4.42E-02 |
| plasma | B | GP5 | -0.6885 | 0.1532 | 6.96E-06 | 1.23E-04 |
| plasma | B | GP10 | -0.5261 | 0.1452 | 2.90E-04 | 3.24E-03 |
| plasma | B | GP27 | 0.6267 | 0.1830 | 6.17E-04 | 4.89E-03 |
| plasma | B | GP36 | 0.6649 | 0.1950 | 6.52E-04 | 4.93E-03 |
| plasma | B | GP35 | 0.5633 | 0.1795 | 1.70E-03 | 1.04E-02 |
| plasma | B | GP4 | -0.6372 | 0.2071 | 2.09E-03 | 1.16E-02 |
| plasma | B | GP16 | -0.4705 | 0.1549 | 2.39E-03 | 1.21E-02 |
| plasma | B | GP13 | -0.4304 | 0.1566 | 6.01E-03 | 2.60E-02 |
| plasma | B | GP33 | 0.7015 | 0.2618 | 7.38E-03 | 3.07E-02 |
| plasma | B | GP7 | -0.4196 | 0.1611 | 9.20E-03 | 3.55E-02 |
| plasma | B | GP32 | 0.4022 | 0.1615 | 1.27E-02 | 4.43E-02 |
| plasma | A | GP24 + GP25 | -0.4521 | 0.1303 | 5.20E-04 | 4.44E-03 |
| plasma | A | GP33 | 0.4046 | 0.1292 | 1.73E-03 | 1.04E-02 |
| plasma | A | GP27 | 0.3729 | 0.1282 | 3.63E-03 | 1.75E-02 |
| plasma | A | GP31 | -0.3750 | 0.1323 | 4.59E-03 | 2.16E-02 |
| plasma | A | GP26 | -0.3852 | 0.1387 | 5.47E-03 | 2.51E-02 |
| plasma | A | GP39 | 0.4850 | 0.1763 | 5.94E-03 | 2.60E-02 |
| plasma | A | GP30 | -0.3548 | 0.1352 | 8.66E-03 | 3.40E-02 |
| plasma | A | GP35 | 0.3471 | 0.1340 | 9.61E-03 | 3.57E-02 |
| IgG | D | IGP1 | 0.7629 | 0.1579 | 1.35E-06 | 1.78E-04 |
| IgG | D | IGP9 | -0.5823 | 0.1590 | 2.51E-04 | 1.10E-02 |
| IgG | B | IGP9 | -0.5458 | 0.1583 | 5.65E-04 | 1.67E-02 |
| IgG | C | IGP2 | 1.0150 | 0.3069 | 9.43E-04 | 1.78E-02 |
| IgG | C | IGP7 | 0.9281 | 0.3042 | 2.29E-03 | 3.35E-02 |
| IgG | D | IGP4 | 0.4680 | 0.1562 | 2.74E-03 | 3.62E-02 |
| IgG | D | IGP14 | -0.4582 | 0.1549 | 3.10E-03 | 3.72E-02 |
| ***Origin*** | ***ABCD group based on mMRC*** | ***Derived glycan trait*** | ***Beta*** | ***SE*** | ***Meta-analysis p-value*** | ***Meta-analysis adjusted p-value*** |
| plasma | D | G1 | -0.9391 | 0.1530 | 8.31E-10 | 8.81E-08 |
| plasma | D | AntF | 0.8745 | 0.1461 | 2.15E-09 | 1.11E-07 |
| plasma | D | G4 | 0.8638 | 0.1451 | 2.61E-09 | 1.11E-07 |
| plasma | D | S3 | 0.5919 | 0.1566 | 1.57E-04 | 2.08E-03 |
| plasma | D | S0 | -0.7453 | 0.2113 | 4.20E-04 | 4.05E-03 |
| plasma | D | S4 | 0.8091 | 0.2365 | 6.22E-04 | 4.89E-03 |
| plasma | D | LB | -0.4890 | 0.1583 | 2.01E-03 | 1.15E-02 |
| plasma | D | HB | 0.4844 | 0.1581 | 2.19E-03 | 1.16E-02 |
| plasma | D | OligoMann | -0.3916 | 0.1569 | 1.25E-02 | 4.43E-02 |
| plasma | C | G4 | 1.0016 | 0.2774 | 3.06E-04 | 3.24E-03 |
| plasma | C | AntF | 0.9277 | 0.2790 | 8.85E-04 | 6.08E-03 |
| plasma | C | S4 | 0.8441 | 0.2874 | 3.31E-03 | 1.63E-02 |
| plasma | B | G1 | -0.6609 | 0.1552 | 2.05E-05 | 3.35E-04 |
| plasma | B | S3 | 0.6305 | 0.1588 | 7.19E-05 | 1.08E-03 |
| plasma | B | S0 | -0.5487 | 0.1582 | 5.24E-04 | 4.44E-03 |
| plasma | B | HB | 0.5171 | 0.1605 | 1.27E-03 | 8.40E-03 |
| plasma | B | LB | -0.5112 | 0.1606 | 1.46E-03 | 9.37E-03 |
| plasma | B | CoreF | -0.4869 | 0.1588 | 2.17E-03 | 1.16E-02 |
| plasma | B | AntF | 0.6832 | 0.2635 | 9.53E-03 | 3.57E-02 |
| plasma | A | G4 | 0.4296 | 0.1293 | 8.90E-04 | 6.08E-03 |
| IgG | D | G1 | -0.6090 | 0.1573 | 1.08E-04 | 7.15E-03 |
| IgG | B | G1 | -0.5354 | 0.1567 | 6.32E-04 | 1.67E-02 |
| IgG | C | CoreF | -1.0177 | 0.3076 | 9.37E-04 | 1.78E-02 |
| IgG | D | G0 | 0.4816 | 0.1555 | 1.96E-03 | 3.23E-02 |

*AntF – antennary fucosylation; beta - standardized regression coefficient; COPD – chronic obstructive pulmonary disease; CoreF – core fucosylation; G1 – monogalactosylation; G4 – tetragalactosylation; GP – plasma glycan peak; HB – high branching; IGP – IgG glycan peak; LB – low branching; mMRC – Modified Medical Research Council questionnaire; OligoMann – oligomannosylation; S0 – asialylation; S3 – trisialylation; S4 – tetrasialylation; SE- standard error.
