# Supplementary material for: N-glycosylation patterns of plasma proteins and immunoglobulin G in chronic obstructive pulmonary disease
Source: J Transl Med. 2018 Nov 21;16:323. doi: 10.1186/s12967-018-1695-0 (PMC6249776; doi:10.1186/s12967-018-1695-0)
Supplement: Supplementary file 6 — Additional file 6: Table S5. Associations of plasma glycan traits with the exacerbation frequency (cases with different occurrence of exacerbation events vs healthy controls). Just the glycan traits with statistically significant associations are presented, resulting from case-control meta-analysis. Glycan data were adjusted for age and sex, and corrected for multiple comparisons (Benjamini-Hochberg method). [file 12967_2018_1695_MOESM6_ESM.docx]

Additional file 6: Table S5. Associations of plasma glycan traits with the with exacerbation frequency (cases with different occurrence of exacerbation events vs healthy controls). Just the glycan traits with statistically significant associations are presented, resulting from case-control meta-analysis. Glycan data were adjusted for age and sex, and corrected for multiple comparisons (Benjamini-Hochberg method).*

| ***Exacerbations in the past 12 months*** | ***Glycan*** | ***Beta*** | ***SE*** | ***Meta-analysis p-value*** | ***Meta-analysis adjusted p-value*** |
| --- | --- | --- | --- | --- | --- |
| 2 | GP4 | -0.6972 | 0.1952 | 3.54E-04 | 2.02E-02 |
| 2 | GP6 | -0.6661 | 0.1931 | 5.60E-04 | 2.02E-02 |
| 2 | GP14 | 0.6667 | 0.1953 | 6.43E-04 | 2.02E-02 |
| 2 | GP5 | -0.6319 | 0.1923 | 1.02E-03 | 2.15E-02 |
| 2 | GP29 | 0.6086 | 0.1992 | 2.24E-03 | 3.40E-02 |
| ***Exacerbations in the past 12 months*** | ***Derived glycan trait*** | ***Beta*** | ***SE*** | ***Meta-analysis p-value*** | ***Meta-analysis adjusted p-value*** |
| 2 | G1 | -0.6535 | 0.1942 | 7.63E-04 | 2.02E-02 |
| 2 | S0 | -0.6099 | 0.1959 | 1.85E-03 | 3.26E-02 |

* beta - standardized regression coefficient; G1 – monogalactosylation; GP – plasma glycan peak; S0 – asialylation; SE- standard error.
