# Supplementary material for: N-glycosylation patterns of plasma proteins and immunoglobulin G in chronic obstructive pulmonary disease
Source: J Transl Med. 2018 Nov 21;16:323. doi: 10.1186/s12967-018-1695-0 (PMC6249776; doi:10.1186/s12967-018-1695-0)
Supplement: Supplementary file 7 — Additional file 7: Table S6. Associations of plasma and IgG glycan traits with the smoking status (smokers / ex-smokers vs non-smokers). Just the glycan traits with statistically significant associations are presented, resulting from case-control meta-analysis. Glycan data were adjusted for age and sex, and corrected for multiple comparisons (Benjamini–Hochberg method). [file 12967_2018_1695_MOESM7_ESM.docx]

Additional file 7: Table S6. Associations of glycan traits with the smoking status (smokers / ex-smokers vs non-smokers). Just the glycan traits with statistically significant associations are presented, resulting from case-control meta-analysis. Glycan data were adjusted for age and sex, and corrected for multiple comparisons (Benjamini-Hochberg method).*

| ***Origin*** | ***Smoking status*** | ***Derived glycan trait*** | ***Beta*** | ***SE*** | ***Meta-analysis p-value*** | ***Meta-analysis adjusted p-value*** |
| --- | --- | --- | --- | --- | --- | --- |
| plasma | current | G1 | -0.7133 | 0.1120 | 1.93E-10 | 6.82E-09 |
| plasma | current | CoreF | -0.6658 | 0.1128 | 3.61E-09 | 6.38E-08 |
| plasma | current | S0 | -0.6562 | 0.1138 | 8.06E-09 | 1.22E-07 |
| plasma | current | G4 | 0.5982 | 0.1055 | 1.42E-08 | 1.67E-07 |
| plasma | current | AntF | 0.5830 | 0.1055 | 3.26E-08 | 3.46E-07 |
| plasma | ex | G1 | -0.6224 | 0.1276 | 1.07E-06 | 7.09E-06 |
| plasma | current | S3 | 0.5152 | 0.1127 | 4.79E-06 | 2.82E-05 |
| plasma | ex | S0 | -0.5667 | 0.1293 | 1.16E-05 | 5.36E-05 |
| plasma | ex | CoreF | -0.5546 | 0.1282 | 1.53E-05 | 6.74E-05 |
| plasma | current | G0 | -0.4574 | 0.1091 | 2.75E-05 | 1.16E-04 |
| plasma | current | LB | -0.4022 | 0.1154 | 4.90E-04 | 1.86E-03 |
| plasma | current | HB | 0.3970 | 0.1151 | 5.63E-04 | 2.06E-03 |
| plasma | current | S2 | 0.3999 | 0.1166 | 6.03E-04 | 2.10E-03 |
| plasma | ex | AntF | 0.5187 | 0.1539 | 7.50E-04 | 2.41E-03 |
| plasma | current | S4 | 0.3652 | 0.1124 | 1.16E-03 | 3.52E-03 |
| plasma | ex | G4 | 0.5149 | 0.1684 | 2.23E-03 | 6.38E-03 |
| plasma | ex | S3 | 0.3906 | 0.1289 | 2.44E-03 | 6.81E-03 |
| plasma | current | G2 | 0.3270 | 0.1123 | 3.59E-03 | 9.51E-03 |
| plasma | ex | G2 | 0.3450 | 0.1281 | 7.09E-03 | 1.79E-02 |
| plasma | ex | G0 | -0.3328 | 0.1243 | 7.42E-03 | 1.83E-02 |
| plasma | ex | S2 | 0.3167 | 0.1323 | 1.67E-02 | 3.68E-02 |
| IgG | current | Bisecting | 0.7375 | 0.1241 | 2.78E-09 | 1.83E-07 |
| IgG | current | CoreF | -0.4706 | 0.1333 | 4.16E-04 | 5.50E-03 |
| ***Origin*** | ***Smoking status*** | ***Glycan*** | ***Beta*** | ***SE*** | ***Meta-analysis p-value*** | ***Meta-analysis adjusted p-value*** |
| plasma | current | GP5 | -0.7612 | 0.1109 | 6.70E-12 | 7.11E-10 |
| plasma | current | GP27 | 0.6604 | 0.1009 | 5.88E-11 | 3.12E-09 |
| plasma | current | GP33 | 0.6446 | 0.1029 | 3.69E-10 | 9.77E-09 |
| plasma | current | GP4 | -0.7004 | 0.1159 | 1.49E-09 | 3.16E-08 |
| plasma | current | GP35 | 0.6096 | 0.1070 | 1.23E-08 | 1.63E-07 |
| plasma | current | GP16 | -0.5691 | 0.1066 | 9.32E-08 | 8.98E-07 |
| plasma | ex | GP27 | 0.5989 | 0.1164 | 2.66E-07 | 2.35E-06 |
| plasma | current | GP39 | 0.5331 | 0.1047 | 3.56E-07 | 2.69E-06 |
| plasma | ex | GP4 | -0.6483 | 0.1271 | 3.37E-07 | 2.69E-06 |
| plasma | current | GP14 | 0.5799 | 0.1143 | 3.94E-07 | 2.78E-06 |
| plasma | current | GP1 | -0.4950 | 0.1079 | 4.45E-06 | 2.78E-05 |
| plasma | current | GP10 | -0.4597 | 0.1014 | 5.77E-06 | 3.22E-05 |
| plasma | ex | GP33 | 0.5353 | 0.1186 | 6.39E-06 | 3.39E-05 |
| plasma | current | GP20+GP21 | 0.5069 | 0.1150 | 1.04E-05 | 5.08E-05 |
| plasma | ex | GP35 | 0.5424 | 0.1231 | 1.05E-05 | 5.08E-05 |
| plasma | ex | GP14 | 0.5080 | 0.1292 | 8.45E-05 | 3.44E-04 |
| plasma | ex | GP5 | -0.6004 | 0.1663 | 3.07E-04 | 1.20E-03 |
| plasma | ex | GP10 | -0.4041 | 0.1180 | 6.14E-04 | 2.10E-03 |
| plasma | current | GP36 | 0.3773 | 0.1113 | 7.04E-04 | 2.33E-03 |
| plasma | ex | GP20+GP21 | 0.4273 | 0.1304 | 1.05E-03 | 3.26E-03 |
| plasma | current | GP32 | 0.3489 | 0.1141 | 2.23E-03 | 6.38E-03 |
| plasma | current | GP8 | 0.3401 | 0.1167 | 3.57E-03 | 9.51E-03 |
| plasma | ex | GP16 | -0.3586 | 0.1260 | 4.44E-03 | 1.15E-02 |
| plasma | ex | GP1 | -0.3201 | 0.1228 | 9.13E-03 | 2.20E-02 |
| plasma | current | GP7 | -0.2884 | 0.1154 | 1.25E-02 | 2.94E-02 |
| plasma | current | GP31 | -0.2662 | 0.1089 | 1.45E-02 | 3.34E-02 |
| plasma | ex | GP36 | 0.3069 | 0.1274 | 1.60E-02 | 3.60E-02 |
| plasma | current | GP13 | -0.4400 | 0.1922 | 2.21E-02 | 4.78E-02 |
| IgG | current | IGP10 | 0.6641 | 0.1294 | 2.89E-07 | 9.55E-06 |
| IgG | current | IGP6 | 0.5015 | 0.1260 | 6.94E-05 | 1.53E-03 |
| IgG | current | IGP23 | -0.4998 | 0.1313 | 1.40E-04 | 2.31E-03 |
| IgG | current | IGP15 | 0.4561 | 0.1334 | 6.29E-04 | 6.92E-03 |
| IgG | current | IGP9 | -0.6779 | 0.2270 | 2.82E-03 | 2.32E-02 |
| IgG | current | IGP13 | 0.3933 | 0.1312 | 2.72E-03 | 2.32E-02 |
| IgG | current | IGP2 | 0.3916 | 0.1329 | 3.21E-03 | 2.35E-02 |
| IgG | current | IGP7 | 0.3670 | 0.1325 | 5.61E-03 | 3.48E-02 |
| IgG | current | IGP12 | 0.3639 | 0.1319 | 5.80E-03 | 3.48E-02 |
| IgG | ex | IGP17 | 0.3794 | 0.1438 | 8.34E-03 | 4.59E-02 |

*AntF – antennary fucosylation; beta - standardized regression coefficient; CoreF – core fucosylation; G0 – agalactosylation; G1 – monogalactosylation; G2 – digalactosylation; G4 – tetragalactosylation; GP – plasma glycan peak; HB – high branching; IGP – IgG glycan peak; LB – low branching; S0 – asialylation; S2 – desialylation; S3 – trisialylation; S4 – tetrasialylation; SE- standard error.
